# Push-Out Bond Strength of Calcium Silicate-Based Cements in the Presence or Absence of a Smear Layer

**DOI:** 10.1155/2022/7724384

**Published:** 2022-07-19

**Authors:** Ahmad S. Al-Hiyasat, Wafa'a A. Yousef

**Affiliations:** Department of Conservative Dentistry, Faculty of Dentistry, Jordan University of Science and Technology, Irbid, Jordan

## Abstract

**Objectives:**

Variations between the material compositions and the presence of a smear layer on the dentin surface may influence the bond strength of the material, thus this study evaluated the push-out bond strength of different calcium silicate materials to root dentin in the presence or absence of smear layer.

**Materials and Methods:**

The palatal canal of sixty maxillary first premolars were prepared and divided into three groups according to the materials to be used: ProRoot WMTA, Biodentine, and TotalFill FS. Each group was then divided into two subgroups; with and without a smear layer. Roots were sectioned into three slices and filled with the tested materials. Push-out bond strength of materials was measured by universal machine.

**Results:**

Bond strength of Biodentine was significantly higher than the MTA and TotalFill FS in the presence or absence of smear layer. Overall, removing the smear layer reduced the bond strength of the three materials. The reduction was significant for MTA and TotalFill FS but not for Biodentine.

**Conclusions:**

Biodentine demonstrated the highest bond strength to radicular dentin followed by MTA, and then TotalFill FS. Overall, removal of the smear layer from dentin surface reduced the bond strength of the calcium silicate root repair materials.

## 1. Introduction

The function of root canal obturation is to fill the canal space and eliminate any possible communication between the canal and periodontium [1]. Awareness of the presence of undesirable conditions such as perforations, resorption, or open apices lead to difficulty in achieving a fluid tight seal using the traditional obturation material (gutta percha with a sealer). Thus, there is a need for a reparative material that should adhere well to root canal dentin and maintain the integrity of material-dentin interface under static conditions and to resist displacement of the material during function and operative procedures.

Mineral trioxide aggregate (MTA) is considered the gold standard material for such clinical procedures, as perforation repair, resorption repair, revascularization, apical plug in open apices, retrograde filling in apical surgery, and in vital pulp therapy [2–6]. It is a calcium silicate material with adequate physical and chemical properties [7], low cytotoxicity [8], and good surface for cellular attachment [9]. However, it has long setting time [7], discoloration of the tooth [10], difficult handling [7], and inability to penetrate the dentinal tubules [11].

Biodentine is another calcium silicate material that has been introduced. It has shorter setting time with a less discoloration effect and ability to form tag-like structures extending into the dentinal tubules [10, 12, 13]. But it comes in a preset quantity (capsule), making waste inevitable in the majority of cases [14].

Recently, a new calcium silicate-based bioceramic material named TotalFill Putty became available. It is a premixed bioceramic material with biological properties [15], it contains nano-particles and its manufacturer claims that it is highly resistant to washout and ideal for all types of root repair and pulp capping treatments [16–18]. More recently, a new version Fast Set Putty (TotalFill FS) has been introduced into the market and has all the properties of the original putty but with a faster setting time of only 20 min [16].

The bond of the materials to the dentin depends to the properties of the materials but could also be affected by the present of smear layer on the dentin surface. Therefore, the objective of this in vitro study was to evaluate and compare the push-out bond strength of MTA, Biodentine, and TotalFill FS in the presence or absence of a smear layer from the root dentin surface.

## 2. Materials and Methods

### 2.1. Teeth Selection and Preparation

Sixty maxillary first premolars that were freshly extracted for orthodontic reasons were cleaned and stored in distilled water. The crowns were reduced to achieve a standard length of 20 ± 1 mm. After access cavity, the palatal root canals were instrumented with ProTaper files up to F5 (Dentsply, Maillefer, Ballaigues, Switzerland) to a length of 5 mm beyond the apex to achieve a larger diameter canal, 20 ml of 5.25% NaOCl were used as irrigation solution. Teeth were then divided randomly into 3 groups (*n* = 20) according to the materials to be tested, and each group was then divided into 2 subgroups: A1, B1, and C1 with a smear layer maintained, while in A2, B2, and C2, the smear layer was removed by 1 ml of 17% ethylenediaminetetraacetic acid (EDTA) for 1 min followed by 2 ml of 5.25% NaOCl [19]. The root of each tooth was then imbedded into a cold curing acrylic mold, and the apical 3 mm of each root was sectioned and discarded using a water-cooled diamond wheel on “IsoMet 1000 precisions saw” (Buehler, Lake Bluff, IL, USA). Each root was then sectioned at 3 different levels, namely: apical, middle, and coronal to obtain 3 slices of 3 ± 0.2 mm in thickness, the rest of the root with remnant part of the crown were discarded.

### 2.2. Material Preparation and Placement

Root canal sections were filled accordingly with the tested materials, namely, ProRoot WMTA, Biodentine, and TotalFill FS (1) using hand pluggers of different sizes (0.9, 0.7, 0.5 mm) on a glass slap. ProRoot WMTA and Biodentine materials were mixed according to manufacturer's instructions, while TotalFill FS, is a premixed material. Filled sections were stored in cuvettes with cotton soaked with distilled water wrapping each section. Samples were kept in an incubator at 37°C in 100% humidity for 48 hours for final setting of the materials.

The greater (coronal) and lesser (apical) canal diameter for each section was recorded using a digital caliper (Vogal, Kevelaer, Germany), and the section thickness was measured using a metal gauge (I wanson Spring Caliper for Metal, Hu-Friedy, Germany). The adhesion surface area was calculated by the following equation:

(1) $$\begin{array}{c} \text{Adhesion surface area}\left(mm{}^{2}\right)=\left(\frac{D1+\;D2}{2}\right)\times µ\times h, \end{array}$$

where D1 and D2 are the greater and lesser canal diameter, respectively, *μ* is the constant 3.14, and *h* is the height, which represents the thickness of the root section.

### 2.3. Push-Out Test

**Figure 1:**
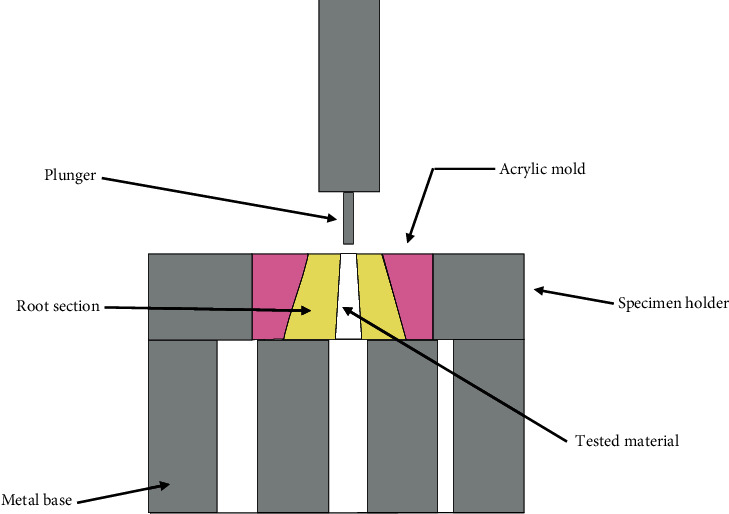
Illustration for the push-out test of the material tested from the root canal section.

The force required to dislodge the material from the canal was measured using a universal testing machine (Jinan Testing Equipment IE corporation, China). A metal base was made with 3 holes of different sizes as a platform to place the sample on it, with specimen holder to hold the root section on the metal base. Plungers with 3 different diameters (1 mm, 0.7 mm, 0.5 mm) corresponding to the coronal, middle, and apical section, respectively, were used to push the material out of the canal. Vertical load was applied over the filling materials in apical-coronal direction with a crosshead speed of 0.1 mm/minutes (Figure 1) [20].

The maximum force in Newton (F-max) at which the dislodgement of materials occurred was recorded, and the push-out bond strength in megapascal (MPa) was calculated for each sample according to the following equation:

Push-out bond strength (MPa) = F-max/adhesion surface area (mm^2^), where F-max: Maximum force.

The data for each sample in each group were registered for statistical analysis.

### 2.4. Mode of Failure

After the push-out test, root canal sections were examined by an optical microscope at 40x (Olympus, Tokyo, Japan) to determine the bond failure mode which was classified into the following:

- Adhesive: failure was at the material-dentin interface (dentine surface without material)
- Cohesive: failure was entirely within the material (dentin surface totally covered by material)
- Mixed: adhesive and cohesive modes (some material left attached to the dentin surface).

### 2.5. Scanning Electron Microscopy (SEM)

Two samples from each group were picked randomly after the push-out test as representative of each group. The samples were split along the center of the canal having 2 halves from each sample totaling 4 specimens for each group. The pulpal wall of the root canal was sputter-coated with gold (Q150R ES sputter coater, Quorum Technologies, United Kingdom) and examined under SEM (Quanta FEG 450, FEI, Netherlands).

### 2.6. Statistical Analysis

The data of the push-out test were statistically analyzed by ANOVA analysis of variance-operated by Minitab statistical package. Two-way ANOVA was used to determine the statistical and significant effect of the study variables (materials type and smear layer), followed by a Tukey pairwise comparison test at 95% confidence intervals.

## 3. Results

### 3.1. Push-Out Test

Since the purpose of this study was to compare the bond strength of the three materials to the root dentin, the data of the three-root sections were pooled together so it will present the bond of the materials at the different levels of the root canal dentin.

**Figure 2:**
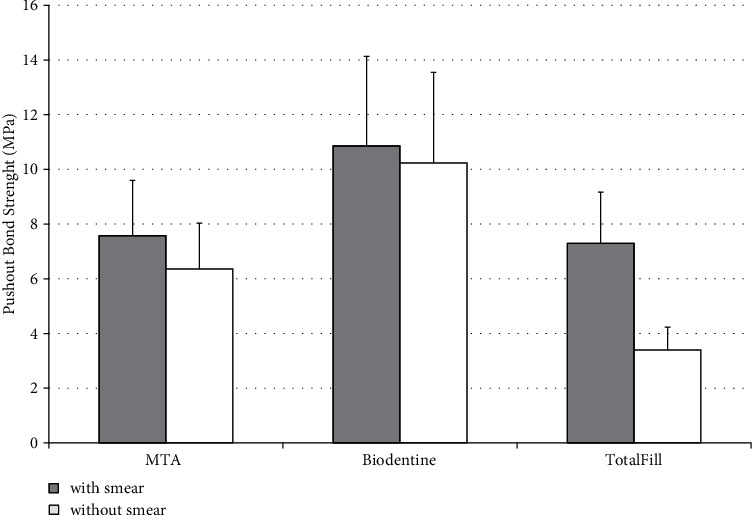
Means of the push-out bond strength (MPa) of the three materials tested with and without smear layer on the dentin surface (error bar represents SD).

The results showed that with or without a smear layer, Biodentine had the highest bond strength to dentin, followed by MTA, and the weakest bond was with TotalFill FS (Figure 2). Overall, Figure 2 shows that for the three materials the bond was reduced when the smear layer was removed. This was more obvious with TotalFill FS in which the mean value of the bond strength decreased by more than 50%.

Two-way analysis of variance (ANOVA) revealed that both, materials and smear layer had a highly significant effect on the bond strength (*P* < 0.001) as well as the interaction (*P* < 0.001). Therefore, the data were further analyzed to compare the bond strength of the materials, first in the presence of smear layer and then in its absence by one-way ANOVA followed by Tukey test at 95% confidence intervals. The analysis showed that in the smear layer groups, the bond strength of Biodentine (10.85 ± 3.28 MPa) was significantly higher than the bond strength of MTA (7.58 ± 2.02 MPa) and TotalFill FS (7.30 ± 1.87 MPa) (*P* < 0.05). But, the difference between MTA and TotalFill FS was not statistically significant (*P* > 0.05). While in the groups where the smear layer was removed, the differences between the three groups; MTA (6.36 ± 1.68 MPa), Biodentine (10.24 ± 3.31 MPa), and TotalFill FS (3.40 ± 0.83 MPa) were statistically significant (*P* < 0.05). Furthermore, the data of each material in the present of smear layer were compared to the data of the same materials but after the removal of smear layer. The Tukey test showed that the reduction in the bond strength after the removal of the smear layer for the MTA and TotalFill FS was statistically significant (*P* < 0.05), while for the Biodentine, it was not significant (*P* > 0.05) (Figure 2 and Table 2).

### 3.2. Mode of Failure

The optical microscope revealed variation on the mode of failure of the materials bond. The percentages of the mode of failure for the three materials in the presence or absence of a smear layer are presented in Table 3.

Overall, none of the samples showed pure adhesive failure; the bond in all the samples had failed either cohesive or mixed mode. It is interesting to notice that for Biodentine and TotalFill FS, the majority of the samples had mixed mode of failure whether the smear layer was present or removed (≥60%). However, in the MTA groups, in those samples with smear layer presence, the majority had cohesive failure (77%) while in those without a smear layer, the majority had mixed mod of failure (63.6%).

### 3.3. Scanning Electron Microscopy (SEM)

**Figure 3:**
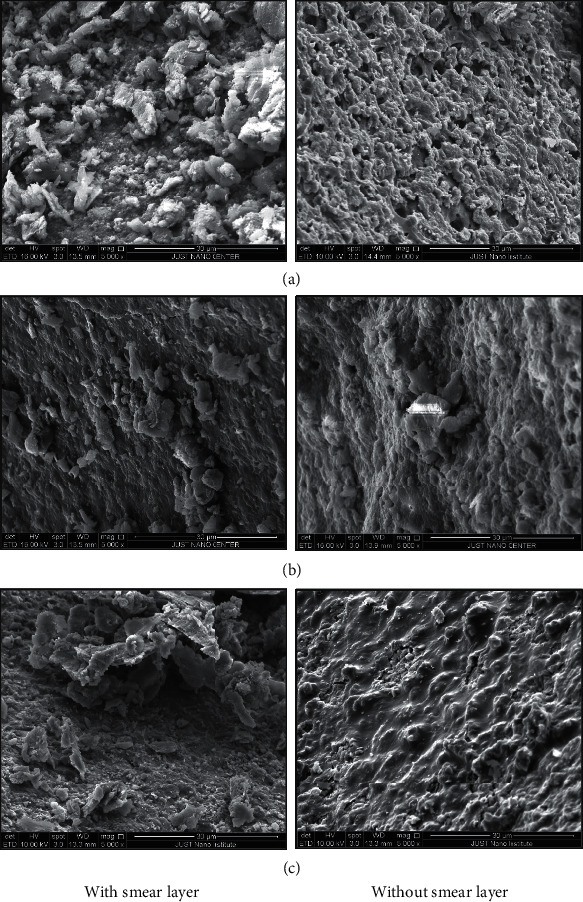
SEM images of root canal dentin surfaces that were filled with; (a) MTA, (b) Biodentine, and (c) TotalFill FS, with a smear layer preserved (left side, with smear layer) and after removal of smear layer (right side, without smear layer).

The samples of the dentin surface with the three materials tested with a smear layer preserved and without a smear layer are presented in Figure 3. It can be observed that in those samples with a preserved smear layer, overall, there are bulks of materials that had been attached to the surface of the dentin, and they are engaged with the smear layer. The bulks were more obvious in MTA and TotalFill FS, in fact in TotalFill FS, it was like a collection of particles that are detached from the surface. However, with Biodentine, the particles were much smaller and more spread and engaged within the surface. While in those samples without a smear layer, we could see that the remnants of the materials were in much smaller particles and were spreading all over the surface in a more uniform texture. In TotalFill FS, the texture was more uniform and had a smooth appearance compared with that of MTA and Biodentine. Furthermore, we could see that the dentinal tubules were clearly opened in MTA and to less extent in the Biodentine where the smear layer was removed (Figures 3(a) and 3(b), right side), while in TotalFill FS, the dentinal tubules were filled with particles of the material that were intruded within the dentinal tubules (Figure 3(c), right side).

## 4. Discussion

Overall calcium silicate-based materials release calcium hydroxide during their hydration reaction [21], and an interfacial layer ‘hydroxyapatite' is formed between root canal dentin and the calcium silicate-based materials [12, 21], thus enhancing the bond at the material-dentin interface.

In this study, the push-out bond strength of Biodentine was significantly higher than that of MTA and TotalFill FS with or without a smear layer. The superiority of the bond strength of Biodentine over the MTA with or without a smear layer has been reported before [22]. This could be attributed to variation of the bioactivity level between the materials tested, which is related to the compositional differences of the materials [23, 24]. The releasing of Ca ions, and apatite-forming ability, with interfacial layer formation are thought to be useful for predicting the bioactivity of the material [25]. It has been shown that Biodentine has a higher bioactivity which might have triggered the formation of tag-like structures at the cement-dentin interface and increased the dislodgement resistance of Biodentine as compared with MTA and the premixed bioceramic “ERRM” [23, 26], Han and Okiji's [26] results demonstrated that Biodentine had significantly more prominent biomineralization ability than MTA. This attributed to the differences in the amount of Ca and Si released; Biodentine released larger amount of Ca and Si than MTA and, consequently, produced larger amounts of calcium phosphate precipitates in the PBS environment. Although the precise role of Si in hard tissue metabolism remains unclear, it was reported that Si induces remineralization of demineralized dentin in vitro [27]. Such a property may have positively influenced the formation of the interfacial layer and the tag-like structure [26]. The same conclusion was observed by the same authors in another study in which the concentration of Ca ions released was in the following order of; Biodentine > MTA > BC sealer “EndoSequence BC sealer” at different time intervals (5 hours–168 hours) after immersion in PBS [23]. The analysis of the material-dentin interface revealed the formation of tag-like structures within dentinal tubules, which composed mainly of Ca and P, in all the materials, and Biodentine showed more prominent Ca and Si incorporation in adjacent to dentin compared with MTA and BC sealer [23]. This further support our results although we used distilled water as an immersion solution that might influenced some of the elements released, a matter which could be consider for further investigation.

The differences in Ca ions released between materials could be related to the differences in their compositions. Camilleri [28] analyzed the composition of ProRoot white MTA and reported that it contains 55% tricalcium silicate and 20% dicalcium silicate. For Biodentine, it has been reported by the same author with co-workers [21] that it contains 80.1% tricalcium silicate and no dicalcium silicate (0%). Regarding TotalFill FS composition, the manufacturer data sheet reported the percentage of tricalcium silicate between 30% - 36% and 9%–13% for the dicalcium silicate [16]. As it is reported and known, the tricalcium silicate is the main phase present in calcium silicate materials and the responsible for Ca ions release [21], this could indicate that Biodentine releases more Ca ions than MTA and TotalFill FS, and thus showed significantly greater bond strength than them.

Furthermore, it has been reported that the adhesion of reparative materials to root canal dentin is influenced by the presence or absence of the smear layer [22]. In our study, it was noticed that the removal of a smear layer significantly decreases the push-out bond strength of all the materials tested. This is in agreement with the findings of El-Ma'aita et al. [22], who reported that there was a consistent decrease in the push-out bond strength of MTA and Biodentine when the smear layer was removed. The author reported that the smear layer was important in the formation of the interfacial layer and possibly gets actively involved in the mineral interaction between calcium silicate cement and radicular dentin [22]. This was obvious in the MTA and TotalFill groups which had significant reductions in the bond strength after the smear layer removal. This could be related to the amount of calcium release from the material which is related to the content of tricalcium silicate in the material composition as it was mentioned before [21].

Although, removal of the smear layer reduced the bond strength of all the materials tested in the present study, Biodentine still demonstrated significantly higher bond strength than MTA and TotalFill FS. This could also be related to the particle size of the materials. The ability of any material to penetrate the dentinal tubules can be attributed to the size of dentinal tubules, and the particle size of the material. Dentinal tubules are structures that range in diameter from 0.9 to 2.5 *μ*m at the pulpal wall [29], so for any material to penetrate the tubules, the particle size must be smaller than the diameter of the tubules. The diameter of particle size for ProRoot MTA is 2.96–2.36 *μ*m [30]. A study using SEM demonstrated that MTA failed to penetrate the dentinal tubules at any level [11]. Therefore, the removal of smear layer may not result in improving the bond of MTA to dentinal wall as would be expected. In fact, removing the smear layer consequently reduced the bond strength to radicular dentine, which could be explained by the interaction that may occur between the particles of MTA and the smear layer. In contrast, Atmeh et al. [12] reported the formation of tag-like structures within the dentinal tubules in Biodentine which was associated with the presence of calcium carbonate that originated from either Ca (OH)_2_ carbonation or its presence within Biodentine. The smaller particle size of Biodentine may be conducive to the formation of tag-like structures and better micromechanical adhesion to dentin [12, 21]. Our finding is in agreement with others who noticed that Biodentine had significantly higher bond strength to radicular dentine than MTA when the smear layer was removed, they related that to the small particle size of Biodentine compared to that of MTA and also the more active interfacial layer with dentine surface [22, 31, 32].

For the Premixed bioceramic TotalFill FS, our results showed that TotalFill FS had significantly lower bond strength than MTA and Biodentine with and without the smear layer. In fact, it showed more reduction in the bond strength than the other two materials after the smear layer was removed. Although, TotalFill FS was made of nanoparticles as the manufacturer claims (1 × 10^−3^ *μ*m) [16], which may make the penetration of the material into the dentinal tubules more possible, as it was seen in SEM, it did not strengthen the bond. So again, the present of smear layer was important in the formation of the interfacial layer [22], which is also related to the content of tricalcium silicate in the material composition [21]. Therefore, TotalFill was the most affected when the smear layer was removed since it has the lowest amount of tricalcium silicate, followed by MTA, and the least that was affected was the Biodentine which has the highest amount of tricalcium silicate compared to the other two materials.

Furthermore, EDTA induces changes in the dental tissue structure and in calcium and phosphorus ion levels in the dentin [33]. This may cause difficulty in the adaptation of root canal reparative materials to root canal wall, thus lowering bond strength [34–37]. Ari and Erdemir [36] showed that there was a significant decrease in the calcium and phosphorus levels in dentin after the use of EDTA, which is in agreement with the Dogan and Calt study [37] which showed that 17% EDTA combined with 2.5% NaOCl irrigation as final flush altered the mineral contents of root dentin, and changed the Ca/P ratio of root dentin significantly. Thus, the use of EDTA could affect the interaction between Ca and P, the precipitate formation, and the interaction of calcium silicate-based materials to dentin surface. This could explain why the bond strength was reduced significantly after the removal of smear layer for all the three materials tested.

Mixed mode of failure was the most dominant in all the groups of material except in the MTA group where the smear layer was preserved in which the cohesive failure was the most. The mixed mode of failure means that the adhesion of the materials was varying within areas of the root canal. In some area, the material was able to adhere quit well, and in some, it was not. This could be in relation to the presence or absence of the smear layer on root dentine in those groups where the EDTA was used, in which most probably during the irrigation with EDTA; in some areas, the smear layer was removed and the dentine was affected by the EDTA, while in other areas, they were not affected, which reflected in the mixed mode of failure that we noticed. The explanation for those groups where the smear layer was not removed and having a cohesive mode of failure as in MTA group, while a mixed failure in Biodentine and TotalFill FS, this could be due to the porous nature and the granular consistency of MTA which making it more easily engaged within the surface of the smear layer that further become enhanced by the formation of the bioactive surface layer. Thus, breaking this bond was stronger than a break that occurs within the material itself due to its porous nature. However, the consistency of the Biodentine and TotalFill FS was more like a paste; thus, it will be adapted over the surface of the smear layer more easy since they have a smaller particles size than MTA as it was seen in the SEM photos, and due to its paste consistency and the more homogenous mixed the fracture within the material will be less likely to occur, therefore, the mode of failure was more of mixed nature compared to the cohesive failure that was found in the MTA.

So based on the study results, it can be summarized that the variation in the bond strength between calcium silicate-based materials depends on the compositions of the materials, particle size and the formation of a bioactive hydroxyapatite layer on the surface with the dentin, which is related to the content of tricalcium silicate in the material composition. Therefore, Biodentine had the highest bush out bond strength more than the ProRoot MTA and the premixed bioceramic TotalFill FS. Furthermore, removing the smear layer from the root dentin surface reduced the bond strength of three materials tested.

## 5. Conclusion

Within the limitation of this in vitro study and under its condition, the following statements were concluded:

- The push-out bond strength of Biodentine was significantly higher than that of ProRoot MTA and the premixed bioceramic TotalFill FS with or without a smear layer.
- Overall, removing the smear layer reduced the push-out bond strength of the three materials. The reduction was significant for MTA and TotalFill FS, while in Biodentine, it was not significant.

## Figures and Tables

**Table 1:** The manufacturers and compositions of the tested materials.

Material	Manufacturer	Composition^*∗*^
ProRoot WMTA	Dentsply tulsa dental, johnson city, USA	Powder: Tricalcium silicate, dicalcium silicate, bismuth oxide, tricalcium aluminate, calcium sulphate (gypsum), small quantities of SiO_2_, CaO, MgO, K_2_SO_4_, and Na_2_SO_4_. Liquid: Water

Biodentine	Septodont, saint maur des fosses, France	Powder: Tricalcium silicate, dicalcium silicate, calcium carbonate, zirconium oxide, calcium oxide, and iron oxide. Liquid: Calcium chloride, a hydrosoluble polymer, and water

TotalFill FS	Brasseler, dental LLC, savannah, USA distributed by FKG dentaire SA, La-chaux-de-fonds, Switzerland	Premixed: Tricalcium silicate, dicalcium silicate, zirconium oxide, tantalum pentoxide, calcium sulphate (anhydrous), and ﬁllers

^*∗*^Composition of the materials obtained from the manufacturers information.

**Table 2:** Tukey Pairwise comparisons at 95% confidence intervals for the materials tested with the smear layer and without smear layer.

Pairwise comparisons^*∗*^	With a smear layer	Without a smear layer	Pairwise comparisons^*∗*^	With vs. Without a smear layer
MTA vs Biodentine	S	S	MTA	S
MTA vs. TotalFill	NS	S	Biodentine	NS
Biodentine vs. TotalFill	S	S	TotalFill	S

^*∗*^(S: significant; NS: not significant).

**Table 3:** Percentage of mode of failure for the three materials tested from the root dentin surface with (+) and without (−) a smear layer.

Materials	Root dentine surface	Mode of failure		
		Cohesive (%)	Mixed (%)	Adhesive (%)
Biodentine	+ Smear layer	23.3	76.7	0
	− Smear layer	30	70	0

MTA	+ Smear layer	77	23	0
	− Smear layer	36.4	63.6	0

TotalFill FS	+ Smear layer	33.3	66.7	0
	− Smear layer	40	60	0

## Data Availability

All the data of this study that were generated and analysed are included within this article after the statistical analysis, and raw data are available upon request from the corresponding author.

## Abbreviations

MTA: Mineral trioxide aggregate
TotalFill FS: Total fill fast set
EDTA: Ethylenediaminetetraacetic acid
SEM: Scanning electron microscopy.
